# Comparative Neurology of Circadian Photoreception: The Retinohypothalamic Tract (RHT) in Sighted and Naturally Blind Mammals

**DOI:** 10.3389/fnins.2021.640113

**Published:** 2021-05-14

**Authors:** Jens Hannibal

**Affiliations:** Department of Clinical Biochemistry, Bispebjerg Frederiksberg Hospital, University of Copenhagen, Copenhagen, Denmark

**Keywords:** photoreceptors, circadian rhythms, neurotransmitters, entrainment, pupil reflex

## Abstract

The mammalian eye contains two systems for light perception: an image detecting system constituted primarily of the classical photoreceptors, rods and cones, and a non-image forming system (NIF) constituted of a small group of intrinsically photosensitive retinal ganglion cells driven by melanopsin (mRGCs). The mRGCs receive input from the outer retina and NIF mediates light entrainment of circadian rhythms, masking behavior, light induced inhibition of nocturnal melatonin secretion, pupillary reflex (PLR), and affect the sleep/wake cycle. This review focuses on the mammalian NIF and its anatomy in the eye as well as its neuronal projection to the brain. This pathway is known as the retinohypothalamic tract (RHT). The development and functions of the NIF as well as the knowledge gained from studying gene modified mice is highlighted. Furthermore, the similarities of the NIF between sighted (nocturnal and diurnal rodent species, monkeys, humans) and naturally blind mammals (blind mole rats *Spalax ehrenbergi* and the Iberian mole, *Talpa occidentalis*) are discussed in relation to a changing world where increasing exposure to artificial light at night (ALAN) is becoming a challenge for humans and animals in the modern society.

## Introduction

The daily shift between day and night due to the rotation of the earth toward the sun defines the astronomical day of 24 h, which has shaped almost all life forms on the planet. In mammals, light is perceived through the retina and used for image formation primarily based on the classical photoreceptors, rods and cones, and a non-image forming (NIF) system using the photoreceptor, melanopsin (Do and Yau, 2010). The circadian timing system, which is fundamental for survival by driving physiology (hormone secretion, core body temperature, heart rate) and behavior (sleep/wake, eating) into distinct time and periods of the solar cycle, is a major target for the NIF system. The circadian system is orchestrated by a biological “master” clock located in the suprachiasmatic nucleus (SCN) of the hypothalamus and is constituted by approximately 20,000 neurons (Mohawk and Takahashi, 2011). Each individual SCN neuron exhibits a biological clock with an intrinsic period and phase different from other neurons, but their rhythm is coupled to each other to produce a coherent SCN output rhythm. Output signals (neuronal, hormonal) synchronize the peripheral circadian clocks located in the tissues and organs (Yamaguchi et al., 2003; Welsh et al., 2010). The intracellular molecular machinery driving the circadian oscillation in the majority of the SCN neurons consists of interlocked transcriptional and translational feedback loops involving several clock genes and their products (Mohawk and Takahashi, 2011; Takahashi, 2016). However, the endogenous period length of the SCN clock deviates from the astronomical day of exactly 24 h and therefore, the clock needs a daily resetting by light to align the endogenous phase of the clock with the solar cycle, a process known as photoentrainment (Golombek and Rosenstein, 2010). Photoentrainment in mammals is solely dependent on the retina (Nelson and Zucker, 1981). The neuronal pathway transmitting light to the SCN is known as the retinohypothalamic tract (RHT) (Hannibal, 2002). Other NIF functions mediated by the RHT are light suppression of nocturnal melatonin secretion, as well as masking behavior, and regulation of the pupillary reflex (Do and Yau, 2010).

Within the last 20 years, studies of anatomy and physiology using different mammalian species and, in particular, gene modified mice, have shed light on NIF. Through the discovery of specific neurotransmitters (glutamate and pituitary adenylate cyclase activating polypeptide (PACAP) (Hannibal, 2002) and the photoreceptor melanopsin expressed in subpopulations of retinal ganglion cells (mRGCs) constituting the RHT, a fundamental system in the mammalian eye mediating NIF to the brain was characterized (Do and Yau, 2010).

This review will focus on NIF and studies in the circadian photoentrainment, masking behavior and pupillary light reflex in animal models, neuroanatomy, and physiology in gene modified mice lacking elements of the NIF pathway to the brain such as photoreceptors, neurotransmitters, and their receptors. Furthermore, the different aspects of the effects of light on the physiology between sighted (nocturnal and diurnal rodent species, monkeys, humans) and blind mammals (the naturally blind mole rat *Spalax ehrenbergi and* the Iberian mole, *Talpa occidentalis*) will be discussed.

## Non-Image Forming Photoperception (NIF): Light Entrainment; Photic Phase Response Curve (PRC), Masking, and Pupillary Light Reflex (PLR)

The NIF system of the mammalian eye can be considered as an irradiance detector or “light meter” system used for light entrainment of the circadian system, regulation of masking behavior, and the pupillary light reflex (PLR) (La Morgia et al., 2018; Foster et al., 2020). Furthermore, NIF is involved in sleep and core body temperature regulation. When studying the NIF physiology, the focus has mainly been on light entrainment, masking behavior, and PLR.

### Light Entrainment

**FIGURE 1:**
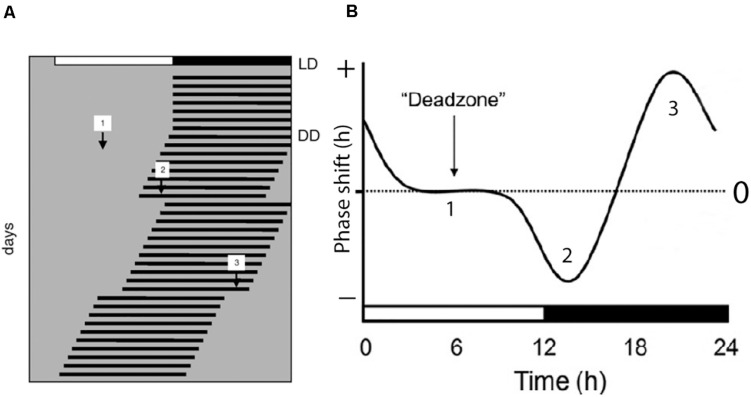
Light regulation of circadian rhythms; the photic phase response curve (PRC). **(A)** Schematic presentation of the activity rhythms of a nocturnal animal where each horizontal line represents the activity of the animal in 1 day. The animal is entrained to a light/dark photoperiod (LD) as represented on the top of the record. The animal is then released into constant darkness (DD) and the activity rhythm is now “free-running.” During the free-running period, the animal experiences light pulses during subjective day [1], early subjective night [2], and late subjective night [3]. The light pulse given during the day has little or no effect on the phase of the endogenous rhythm. Light pulse given in the early subjective night results in a phase delay of the overt rhythm as indicated by [2] in panels **(A,B)**, and a light pulse given in the late subjective night results in a phase advance of the overt rhythm as indicated by [3] in panels **(A,B)**. A complete phase response curve to light stimulation during a 24 h period is drawn in **(B)**. Phase delays are plotted in the negative direction (downward) and phase advances are plotted in the upwards direction. The horizontal axis in **(B)** represents one circadian cycle (from Hannibal, 2020).

Light has a profound effect on the phase of the circadian clock which is fundamental for the ability of the species to stay entrained with the solar cycle. This phase shifting capacity is time dependent which means that light stimulation during daytime in normal feed animals have a little effect on the clock phase while light stimulation during the early part of the subjective night slows down the clock speed by inducing phase delays (Hannibal, 2002). Light stimulation at the end of the night, on the other hand, speeds up the clock resulting in a phase advance of the clock phase (Figure 1). This time dependent effect of light on the clock phase is known as the phase-response curve to light stimulation and is the fundamental ability of the clock to stay entrained with the circadian light/dark cycle as well as the annual cycle (Hughes et al., 2015; Foster et al., 2020). Importantly, light intensity as well as the wavelength and duration of the stimulus determine the size of the phase shift (Hannibal and Fahrenkrug, 2006; Duffy and Czeisler, 2009; Golombek and Rosenstein, 2010).

### Masking

Light can also directly influence the rhythmic physiology and behavior, and this effect is called masking (Mrosovsky, 1999). While nocturnal animals generally become more active in response to darkness (positive masking), light at night has the opposite effect making the animals less active (negative masking). Negative masking causes the inhibition of locomotor activity observed in nocturnal animals when exposed to light at night (Redlin, 2001). While masking is independent of the SCN clock (Redlin and Mrosovsky, 1999), it is an important factor controlling the rhythmic behavior and physiology in arrhythmic animals due to clock gene mutation (Bunger et al., 2000; Husse et al., 2014) or disrupted synchronization of individual clock neurons (Bechtold et al., 2008; Hannibal et al., 2011). A direct inhibition of the nocturnal secretion of the night hormone melatonin can be considered as a “negative” masking by light (Mrosovsky, 1999; Pevet and Challet, 2011).

### Pupillary Light Reflex (PLR)

As early as the late 1920s, Clyde Keeler described a strain of blind house mice which, despite lacking the outer retina, still had light perception and an intact PLR (Keeler, 1924; Keeler et al., 1928). This NIF function was “re-discovered” in the early 1990s by Russell Foster and colleagues (Foster et al., 1991), and the PLR is considered an important parameter when examining the mammalian NIF function. The use of light with different wavelengths can discriminate functional defects located in the outer (i.e., rod and cone defects) or the inner retina (melanopsin defects) (La Morgia et al., 2018).

## The Retino-Hypothalamic Tract (RHT) in Mammals

In 1972, a distinct monosynaptic neuronal pathway to the SCN was described in mammals for the first time (Hendrickson et al., 1972; Moore and Lenn, 1972). These pioneering observations were subsequently confirmed by studies using the subunit B of cholera toxin (CtB) as an anterograde tracer. CtB tracing resulted in a detailed visualization of the RHT projections in several mammalian species (reviewed in Hannibal, 2002). More recently, direct conjugation of different fluorophores to CtB have made it possible to characterize the contralateral and ipsilateral natures of the RHT projections in the mammalian brain into details (Guler et al., 2008; Langel et al., 2015). The RHT originates from a distinct subpopulation of retinal ganglion cells (RGCs) widely distributed in the entire retina (Moore et al., 1995; Hannibal et al., 1997). RHT nerve terminals reach the ventral bilateral SCN (Morin and Allen, 2005), and some of these axons bifurcated and reach the intergeniculate leaflet (IGL) of the lateral geniculate complex which is considered part of the circadian timing system (Pickard, 1985). Nerve fibers from the RHT target several areas in the forebrain which are not directly involved in the circadian timing but are considered as the neuronal pathway of NIF (Canteras et al., 2011). One area, the ventral preoptic area (VLPO), is involved in the homeostatic regulation of sleep (Saper et al., 2005a) and light indirectly affects the hormone secretion dependent of sleep homeostasis (Saper et al., 2005b). Furthermore, RHT directly targets the nerve fibers in the hypothalamic subparaventricular zone (SubPVN), which has been suggested to play a role in light regulated masking behavior (see above) (Shuboni et al., 2012) (see also below). A minor terminal field of the RHT nerve terminals is found in the lateral hypothalamic area dorsal to the supraoptic nucleus (Hannibal and Fahrenkrug, 2004b; Hattar et al., 2006; Canteras et al., 2011). This part of the RHT is supposed to influence the masking effects on specific behaviors such as defensive, drinking, and reproductive behaviors (Swanson, 2000;Canteras et al., 2011).

### The Neuropeptide PACAP: A Marker for the RHT in Mammals

In 1997, a distinct marker labeling all neurons and projections of the RHT to the brain was demonstrated by showing the localization of the neuropeptide PACAP in all RGCs of the rat RHT (Hannibal et al., 1997). The neuropeptide PACAP was discovered in 1989–1990 due to its ability to stimulate cyclic AMP in pituitary cells (Miyata et al., 1989; Miyata et al., 1990). PACAP is, due to its sequence similarities, placed in the family of vasoactive intestinal polypeptide (VIP), secretin and glucagon (Vaudry et al., 2000). PACAP has its own specific receptor, the PACAP type 1 (PAC1) receptor, and shares the VIP types 1 and 2 receptors (VPAC1 and VPAC2) with VIP (Harmar et al., 2012). Retinal PACAP projections densely innervate the bilateral SCN and all other non-visual (NIF) areas of the brain, most intensely the lateral geniculate nucleus and especially the intergeniculate nucleus (IGL) and the ventral geniculate nucleus (VGL), the pretectum including the olivary pretectal nucleus (OPN), and with little innervation of the superior colliculus (SC) (Hannibal and Fahrenkrug, 2004b). The IGL is known as a part of the “circadian visual system” (Morin and Allen, 2005; Morin, 2013), and the OPN is a part of the PLR (Morin, 2013). PACAP immunostaining in combination with CtB injection in the eye has proven useful in the characterization of RHT projections in nocturnal animals such as the rat (Hannibal et al., 1997; Hannibal and Fahrenkrug, 2004b; Engelund et al., 2010), hamster (Bergström et al., 2003), and mouse (Engelund et al., 2012), diurnal rodent (*Arvicantis niloticus*) (Langel et al., 2015), and as well as the monkey (Hannibal et al., 2014). Interestingly, in almost every target area reached by the PACAP nerve terminals, a minor number of retinal projections (CtB positive) not co-storing PACAP were identified. Furthermore, no distinct difference of the melanopsin/PACAP projections were found when comparing the nocturnal and diurnal species (Langel et al., 2015), although, the time of the development of the retinal projections may differ slightly (Todd et al., 2012).

### Identification of Melanopsin in RGCs of the RHT

**FIGURE 2:**
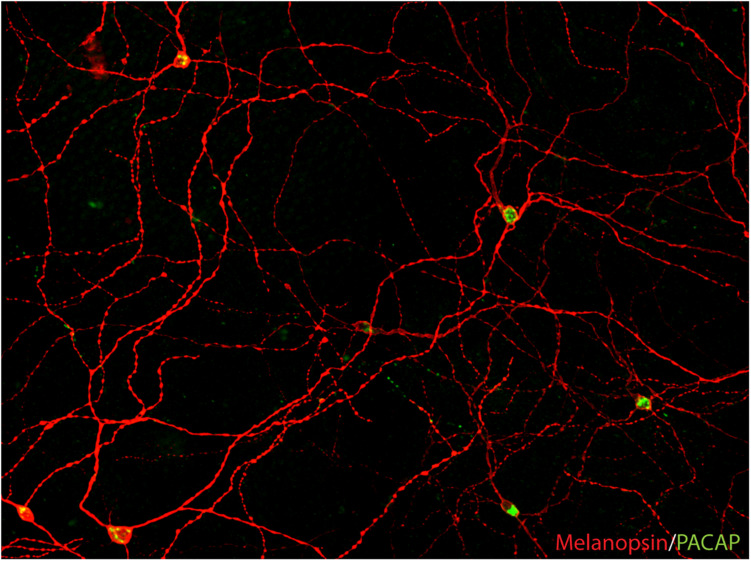
PACAP is found in melanopsin-immunoreactive RGCs in mammals (image from the macaque modified from Hannibal et al., 2014). The image represents a montage of a Z-stack covering the depth required to ensure that both inner and outer stratifying melanopsin processes are visible.

In the 1990s, Russel Foster and colleagues described two mouse models, the rd/rd (rodless/rodless) and the rd/cl (rodless/coneless) mice, characterized by the loss of all rods or both rods and cones during early development (Foster et al., 1991, 1993). Both strains of mice had an intact RHT and were able to photoentrain their circadian rhythm to the light/dark cycle as their littermates (Foster et al., 1991; Freedman et al., 1999). Furthermore, the rd/cl mice photoentrain and suppress nocturnal secretion of melatonin in response to monochromatic light of wavelength of approximately 500 nanometers, indicating that the mammalian retina had additional ocular photoreceptors in the inner retina (Lucas and Foster, 1999; Lucas et al., 1999). In the rd/rd mice with a normal photoentrainment, light induces the transcription factor FOS in neurons of the SCN and in a small population of RGCs (Masana et al., 1996). In rats, light was shown to induce and sustain FOS expression selectively in PACAP expressing RGCs given that the light was ON (both during the subjective day and night). This response was unexpected and unique compared to other RGCs and amacrine cells (in which FOC decreases within 1–2 h) (Hannibal et al., 2001b). This finding was the first direct indication that RGCs of the RHT could be intrinsically photosensitive (Hannibal et al., 2001b). This idea was furthermore supported by the identification of melanopsin, which was initially cloned from frog skin (Provencio et al., 1998). Melanopsin, named from the cells of which it was first isolated (melanophore cells causing the ability of frog skin to shift color), were demonstrated in a small population of RGCs in the mammalian retina shortly after its cloning (Provencio et al., 2000). This observation led to several studies confirming that melanopsin was located exclusively in the surface membrane of RGCs of the RHT (Hannibal et al., 2002a; Hattar et al., 2002; Figure 2). At the same time, the melanopsin-expressing RGCs (mRGCs) were shown to be intrinsically photosensitive (Berson et al., 2002) and shortly hereafter, melanopsin was established as a new mammalian photoreceptor (Berson, 2003; Rollag et al., 2003; Do and Yau, 2010) being sensitive to blue light in a wavelength of approximately 480 nm (Lucas et al., 2014).

Using the melanopsin promoter and knockin of the *tau-lacZ* gene, which codes for the β-galactosidase enzyme fused to a sequence of tau protein and promotes axonal transport of the marker enzyme, Hattar et al. (2002, 2006) were able to visualize the axon projections of the mRGCs (RHT) in the mouse brain, which were very similar to that of the rat (Hattar et al., 2002, 2006; Hannibal and Fahrenkrug, 2004b). Interestingly, using another melanopsin gene (Opn4) knockin (Opn4^*Cre/*+; Z/AP)^ mouse in which CRE-recombinase were combined with an enhanced placental alkaline phosphatase that increases the sensitivity of visualization of melanopsin expressing cells and their projections in the brain, the number of mouse mRGCs were found to be more than twice the number of mRGCs which were initially visualized by immunostaining in mice (Ecker et al., 2010). Brain areas targeted by retinal projections from mRGCs involved in vision processing that were not previously identified [the lateral geniculate complex (LGN) and superior colliculus (SC)], were now identified (Brown et al., 2010; Ecker et al., 2010). These observations were aligned with studies in primates, in which retrograde and anterograde tracing revealed melanopsin projections to both the LGN and SC (Dacey et al., 2005; Hannibal et al., 2014).

### The Diversity of mRGCs

**FIGURE 3:**
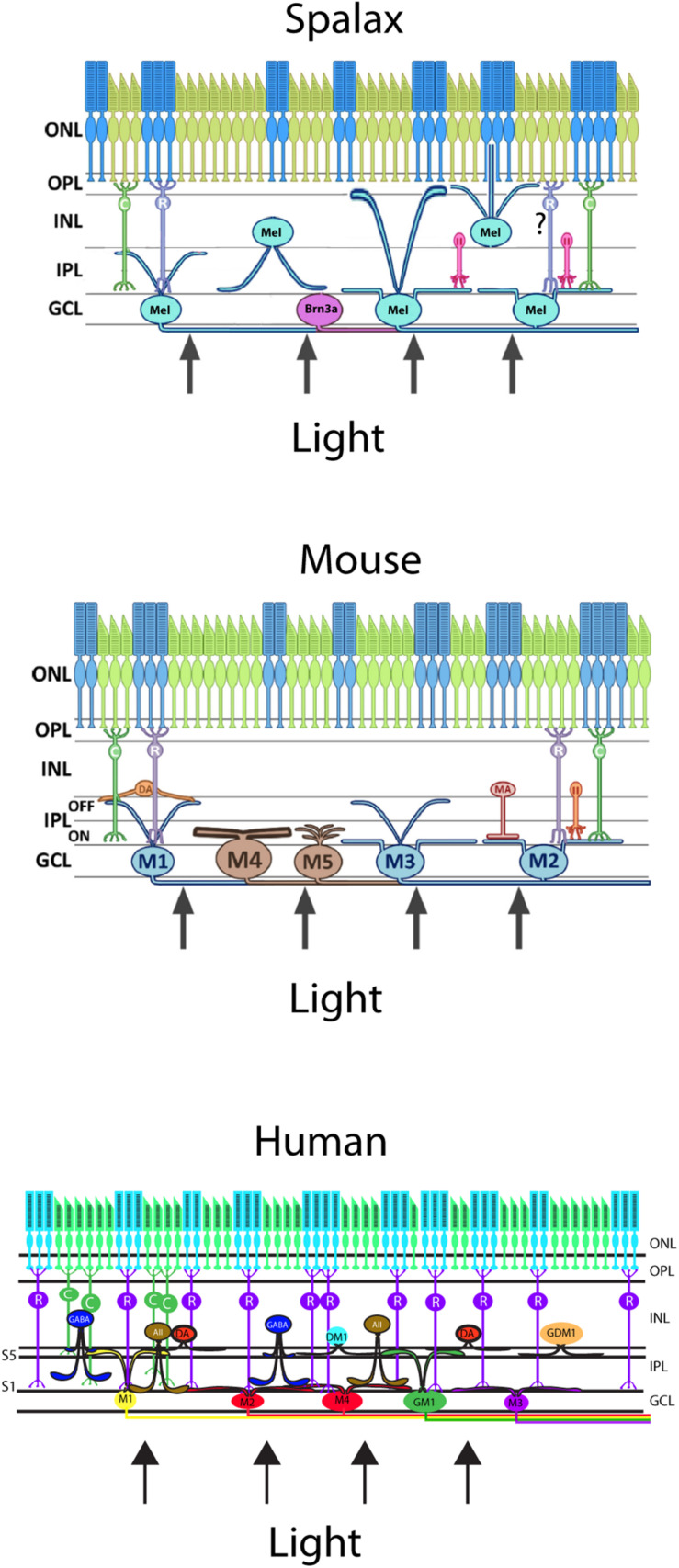
Schematic vertical section of a Spalax (top), mouse (middle), and human (bottom) retina showing the rods and cones (green and blue) and the diverse melanopsin RGC subtypes. Human melanopsin immunoreactive retinal ganglion cells were sub-classified revealing the M1,–displaced M1 (DM1), –M2, –M3, and –M4 cell subtypes and in mouse, the not previously reported gigantic M1 (GM1) and gigantic displaced M1 (GDM1) cells. Melanopsin RGCs receive input from dopamine amacrine cells (DA) and GABAergic amacrine cells found in S5 of the inner plexiform layer (IPL). AII amacrine cell and rod bipolar cell axons innervate the subtypes of melanopsin RGCs located in the ganglion cell layer (GCL). ONL; outer nuclear layer, OPL; outer plexiform layer, INL; inner nuclear layer (modified from Hannibal et al., 2017).

The melanopsin expressing RGCs (mRGCs) were a more heterogeneous group of mRGCs than what was initially suggested and represented by 5–7 subtypes of mRGCs in both rodents and primates, including humans (Baver et al., 2008; Berson et al., 2010; Ecker et al., 2010; Sand et al., 2012; Hannibal et al., 2017). The total number of mRGCs were 0.6–1% of the total number of RGCs in the mammalian retina (Hannibal et al., 2002a, 2017; Hattar et al., 2002). The different subtypes of mRGCs are based on the pattern of dendritic aberration in the inner layers of the inner plexiform layer (IPL) and inner nuclear layer (INL), which form two distinct networks of dendritic projections: one inner stratifying layer located in the sublamina I of the IPL and one outer stratifying layer located in sublaminar V in the IPL close to the INL (Figure 3; Schmidt et al., 2011a; Reifler et al., 2014; Hannibal et al., 2017). These mRGCs differ in the morphology (size, number of dendrites), electrophysical response (Ecker et al., 2010; Schmidt et al., 2011b; Lucas et al., 2014), expression of melanopsin (Pires et al., 2009), and expression of the transcription factor Brn3b (Badea et al., 2009; Chen et al., 2011). The different levels of melanopsin expression in mice seem to be a result of the expression of two isoforms of melanopsin from Opn4 locus, a long isoform (Opn4L) and a short isoform (Opn4S). Both Opn4L and Opn4S are expressed in the mRGCs and Opn4S seems to be 40 times more abundant than Opn4L (Pires et al., 2009). Both isoforms contribute to different functions of the mRGCs (Hughes et al., 2012; Jagannath et al., 2015) (see also below).

### Anatomical Connectivity Between mRGCs and the Outer Retina

In addition to being directly responsive to light, the mRGCs receive light information from the rods and cones as well (Guler et al., 2008). The information is integrated in the mRGCs via bipolar and amacrine cells (Belenky et al., 2003; Jusuf et al., 2007; Ostergaard et al., 2007; Grunert et al., 2011; Lucas et al., 2012; Hannibal et al., 2017). Differences in the dendritic morphology of the mRGCs support the fact that afferent connections differ qualitatively, making the NIF system sensitive to the broad spectrum of light found from sunrise to sunset (Dacey et al., 2005; Lucas et al., 2014).

### Melanopsin in Naturally Blind Mole Rat: The *Spalax ehrenbergi* and the Iberian Mole, the Talpa Occidentalis

**FIGURE 4:**
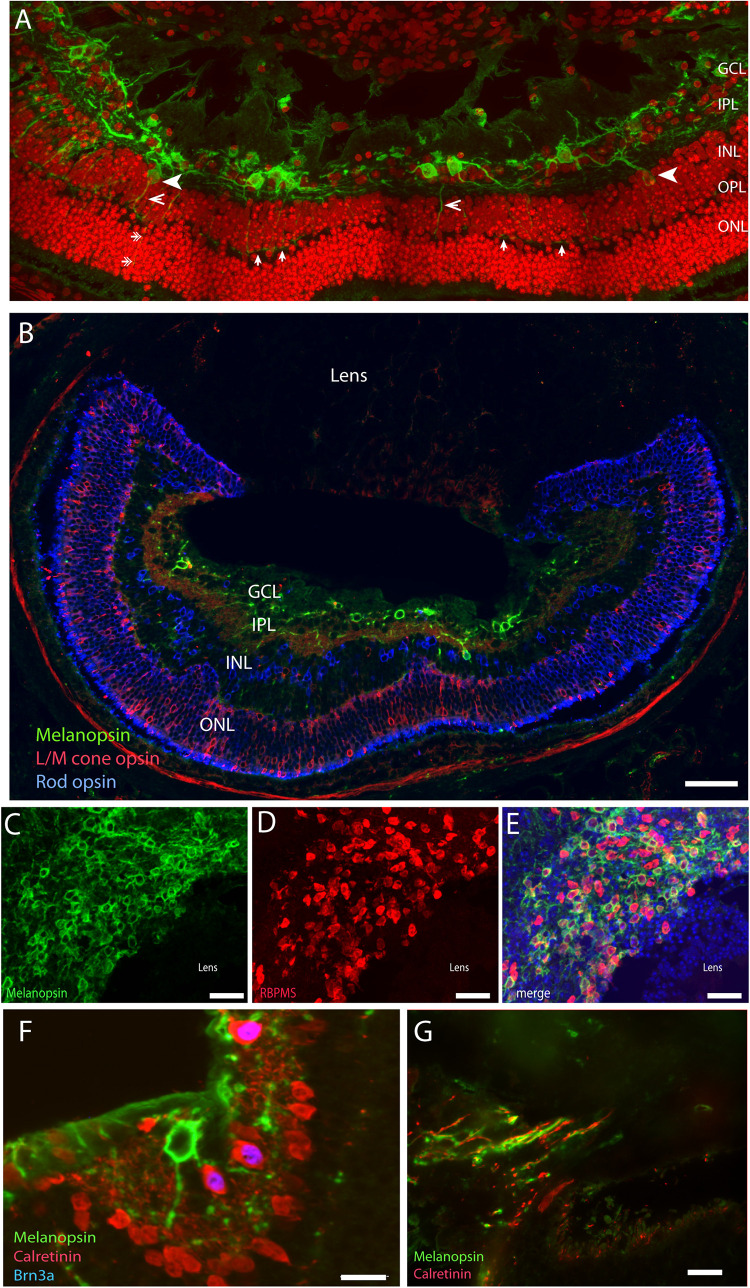
Melanopsin in the naturally blind mole rat, the *Spalax ehrenbergi*. (**A**) Confocal photomicrographs of melanopsin (green) RGC (mRGCs) and DAPI nuclear counterstaining (red) in the *Spalax* retina. Melanopsin RGCs are located in the ganglion cell layer (GCL) and few displaced RGCs are found in the inner nuclear cell layer (INL) (indicated by arrowhead). mRGCs project mainly into the IPL, but also to the outer plexiform layer (OPL) (exemplified by open arrowhead) where they form an outer plexus (indicated by single arrows in **(A)** **(B)** Melanopsin (green), L/M coneopsin (red), and rhodopsin (blue) in *Spalax*. **(C–E)** Ganglion cell marker RBPMS (red) in combination with melanopsin (green) and DAPI nuclear counterstaining (blue) in horizontal sections through the GCL. Brn3a (blue) is found in all non-melanopsin RGCs co-storing calretinin (red) **(F)**. Panel **(G)** shows the optic nerve containing melanopsin and calretinin positive axons. Scalebars: **(A)**: 40 μm, **(B)**: 15 μm, **(C–E)**: 50 μm, **(E,F)**: 15 μm. GCL; ganglion cell layer, IPL; inner plexiform layer, INL; inner nuclear layer (modified from Esquiva et al., 2016).

A large number of mammalian species have adopted a subterranean lifestyle, which in some species have led to natural blindness due to the regression of the eyes and in others, are covered by the skin (Nevo, 1999). Although visually blind, it seems that the eyes of these animals are able to provide information about the circadian and annual cycles (David-Gray et al., 1998; Nevo et al., 2001). The *Spalax ehrenbergi*, which is a blind subterranean mole rat with rudimentary eyes (diameter less than 1 mm) located under the skin, responds to light stimulation and adapts behavior and physiology to both circadian and annual lightnings (David-Gray et al., 1998; Nevo et al., 2001). While the lens of the *Spalax* is pigmented and severely degenerated, the retina is well-organized in the inner and outer layers (Cernuda-Cernuda et al., 2002). The eyes are located in the enlarged harderian gland with an optic nerve that contains less than 900 axons with no image-forming vision (Cooper et al., 1993a). The *Spalax* eye can therefore be considered as a light meter corresponding to the NIF system found in the sighted eye (Cooper et al., 1993b; Hannibal et al., 2002b; Esquiva et al., 2016). Tract tracing from the *Spalax* eye to the brain demonstrates the areas involved in visual perception which receives a significantly reduced retinal projections whereas the brain areas involved in the NIF functions such as the SCN and the ventral geniculate nucleus (VGL) are innervated as found in sighted animals (Bronchti et al., 1991; Cooper et al., 1993b). A detailed study revealed the complex wiring of the classical photoreceptors (rods and L/M cones), amacrine and bipolar cells, and mRGCs, which seem to be similar to that of the sighted mammals (Esquiva et al., 2016; Figure 4). However, while mRGCs represent approximately 1% of all RGCs in sighted animals and humans, nearly 90% of a total of 900 RGCs are mRGCs. As in other mammalian species, mRGCs co-store PACAP, which can be found in retinal target areas in the *Spalax* brain (Hannibal et al., 2002b). Based on the anatomy, the *Spalax* eye seems to represent a functional “light meter” that could resemble what seems to be one of two systems of light perception found in the sighted eye, a system in which classical photoreceptors and mRGCs together constitute the NIF. Interestingly, as found in sighted animals, a minor fraction of *Spalax* RGCs (10%) expressing Brn3a and calretinin, but not melanopsin (Figure 4), projects to the NIF areas in the brain suggesting that non-melanopsin light signaling mediates NIF as well (Esquiva et al., 2016).

The eyes of the Iberian mole, like the *Spalax*, is covered by skin and severely regressed (Carmona et al., 2010). The Iberian mole eye differentiates to form a well-structured retina with all layers as found in a seeing eye [ganglion cell layer (GCL), inner nuclear cell layer (INL), outer nuclear cell layer (ONL), and photoreceptor layer] (Carmona et al., 2010). Although not quantified, a high proportion of RGCs express melanopsin and only a minor part of RGCs express Brn3a (Carmona et al., 2010), as also reported in the *Spalax* (Esquiva et al., 2016).

### mRGCs Are Responsive to Light From Birth

**FIGURE 5:**
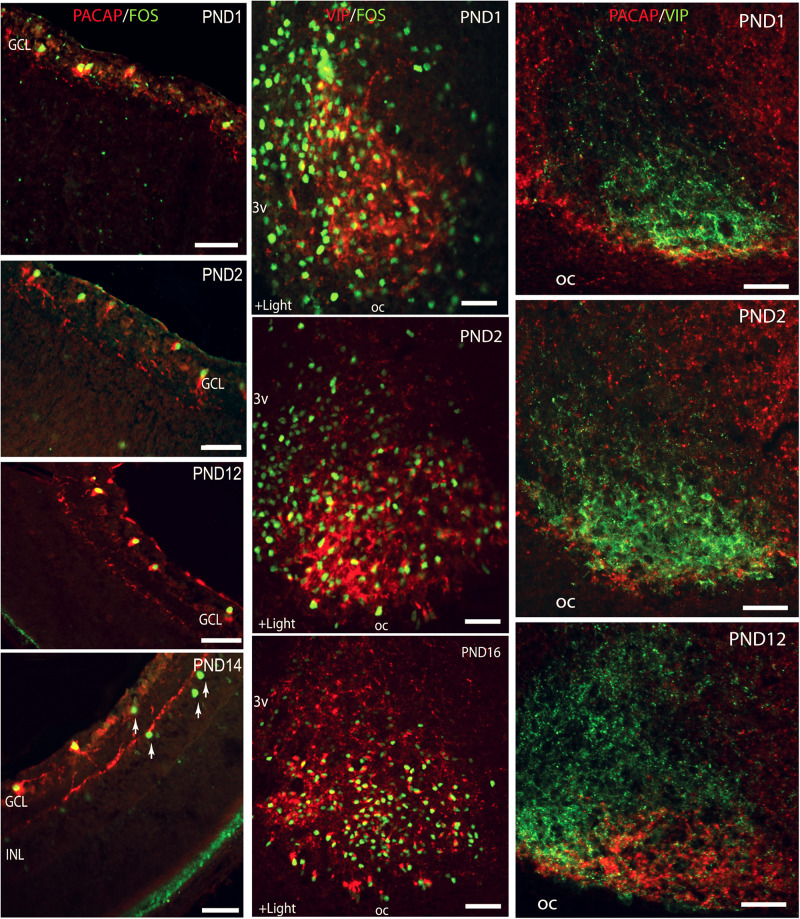
Left lane: Light induction of FOS in melanopsin expressing retinal ganglion cells (RGCs) during postnatal development. Confocal photomicrographs showing double immunostaining for melanopsin (red) and FOS (green) in cross sections of rat retinae obtained from postnatal day (PND1) to PND14. Middle lane: Light stimulation at subjective dawn induces FOS in the SCN from the first postnatal day. Confocal photomicrographs showing double immunostaining for FOS (green) and VIP (red) in sections through the unilateral mid SCN from animals kept in darkness from subjective dawn and receiving light for 1.5 h beginning at subjective dawn. Right lane: Double immunostaining showing postnatal development of the innervation of the ventral SCN (VIP staining in green) by the retinohypothalamic tract (PACAP staining in red). PACAP positive nerve fibers are present in the SCN already on the first postnatal day and an adult pattern is found from PND12. Ages are indicated in each photomicrograph. Oc, optic chiasma. Bars = 50 mm (modified from Hannibal and Fahrenkrug, 2004a).

Melanopsin is expressed in the rat retina from prenatal day 18 (Fahrenkrug et al., 2004), whereas the classical photoreceptors occur and are functional from approximately postnatal day 10, 2–3 days before the eyes open (Ratto et al., 1991). In both rats and mice, NIF is established from P0, a timepoint where light induces FOS in mRGCs and where RHT nerve fibers can be identified in the ventral SCN (Hannibal and Fahrenkrug, 2004a). From this time point, light induces the expression of FOS in the SCN (Hannibal and Fahrenkrug, 2004a; Sekaran et al., 2005; Lupi et al., 2006; Figure 5). Although it has been established that neonatal photoentrainment overrides maternal entrainment from P8, light seems to influence the SCN function from P0 (Takahashi and Deguchi, 1983; Duncan et al., 1986) and may be involved in the development of SCN neuronal networks and retinal pathways (both non-image forming and image forming) in the neonatal brain.

### Melanopsin Expression Is Regulated by Light and the Circadian Clock

Already from birth, the melanopsin expression is regulated both in the pigmented and albino retina, in a process that prenatally are independent of rods and cones (Hannibal et al., 2007). In adults, light has a strong impact on both the melanopsin protein and mRNA expression in the albino retina (Castrucci et al., 2004; Hannibal et al., 2005; Hannibal, 2006a) which seems to override a circadian regulation (Hannibal et al., 2005). In the pigmented retina circadian expression of melanopsin, mRNA seems to be more pronounced than in the albino retina (Sakamoto et al., 2004; Hannibal et al., 2013) and to some extent, dependent of the outer retina and classical photoreceptors (Sakamoto et al., 2004; Wan et al., 2006). However, prolonged exposure to light down regulates the melanopsin protein, whereas prolonged periods of darkness increase the melanopsin protein expression (Hannibal et al., 2013). The functional significance of the melanopsin gene expression regulation remains to be fully clarified but most likely, the changing levels of melanopsin involved in the retinal adaption to environmental light and darkness creating a maximal light sensitivity during the solar cycle.

## Functional Significance of Mrgcs—Learning From the Knockout Models

### mRGC Are the Primary RGCs Mediating NIF

By the discovery of melanopsin in a subpopulation of RGCs making them intrinsically photosensitive (mRGCs), several questions were asked. Is melanopsin the only photoreceptor for the NIF functions? Rod- and cone-less mice had an intact NIF such as photoentrainment, negative masking behavior, and suppression of nocturnal melatonin (Freedman et al., 1999; Lucas and Foster, 1999; Lucas et al., 1999). Generation of melanopsin deficient mice clarified that these mice had a normal circadian rhythm and negative masking compared to their littermates but with a significant blunted phase shift response to nocturnal light pulses indicating an important role of melanopsin in photoentrainment (Panda et al., 2002). Electrophysical examination revealed that that RGCs of melanopsin knockout mice were no longer intrinsically photosensitive (Lucas et al., 2003). These mice have a normal PLR at low light intensities, but the PLR at high light intensity was severely compromised indicating that both the melanopsin and classical photoreceptors were complementary for a normal NIF (Lucas et al., 2003). Mating of melanopsin knockout mice and rodless (rd/rd) mice showed that the double mutant mice lost all the NIF functions [photoentrainment, negative masking behavior, light inhibition of AA-NAT (the enzyme responsible for nocturnal melatonin secretion and prolongation of the endogenous period length during constant light)] (Panda et al., 2003). However, if melanopsin and the classical photoreceptors (rods and cones) mediate NIF to the brain, is it then by a separate neuronal pathway? Anatomically, the mRGCs were identified as the RGCs of the RHT. Tract tracing studies demonstrated that only a few non-melanopsin retinal projections were targeting the NIF areas in the brain innervated by melanopsin neuronal projection (see above). To clarify this question, it was necessary to eliminate the melanopsin expressing RGCs. The Hattar group used the approach to knock-in a gene (aDTA) encoding the attenuated diphtheria toxin A subunit (aDTA) into the mouse gene locus encoding melanopsin (Guler et al., 2008). In the homozygote knock-in mice, an almost complete loss of mRGCs was observed and neurologically, these mice were circadian blind with the loss of all NIF functions while image formation was left intact (Guler et al., 2008). A slightly different approach was used by the Panda group which used a Cre-inducible diphtheria toxin receptor expressed exclusively in the mRGC resulting in a selective loss of mRGCs after the injection of the Diphtheria toxin (DT, which crossed the blood-brain barrier) (Hatori et al., 2008). This approach removed almost all mRGCs and led to a loss of photoentrainment, negative masking behavior, and prolongation of the endogenous period length during constant light as well as the PLR (Hatori et al., 2008). These observations demonstrated that mRGCS are responsible for the NIF function in mammals.

### Different Subtypes of mRGCs Mediate Different Functions

The initial discovery of melanopsin in one subtype of RGCs was modified based on the morphology, expression of melanopsin, dendritic projections/connections, and electrophysical properties. Today, 6–7 subtypes of melanopsin have been identified (Schmidt et al., 2011a; Sand et al., 2012; Reifler et al., 2015; see above and Figure 3). These different subtypes respond to light with slightly different patterns making it likely that they are involved in different functionalities. By dividing mRGCs in M1 and non-M1 subtypes, Chen et al. (2011) found that M1 subtypes can be differentiated based on the expression of Brn3b, a POU domain transcription factor (Badea et al., 2009). Brn3b positive M1 RGCs are functionally different from the Brn3b negative mRGCs. Brn3b-positive mRGCs innervate all other brain targets known to be involved in NIF, including the olivary pretectal nucleus, whereas Brn3b-negative M1 RGCs innervate the suprachiasmatic nucleus (SCN). Selective ablation of Brn3b-positive mRGCs severely disrupts the PLR, but does not impair the circadian photoentrainment consistent with these innervation patterns (Chen et al., 2011). These observations indicate that distinct subpopulations of the M1 subtype of mRGCs innervate different brain regions to execute specific light-induced functions despite being morphologically and electrophysiologically similar. Other melanopsin RGC subtypes (M2, M4, M5, and M6) seem to increase the dynamic range and spectral bandpass of the NIF as well as to shape vision perception (contrast, color, etc.) (Estevez et al., 2012; Weng et al., 2013; Schmidt et al., 2014; Stabio et al., 2018; Quattrochi et al., 2019).

### Different Isoforms of Melanopsin Regulate Different NIF Functions

In mice, melanopsin can be found in a long form (OPN4L) and a short form (OPN4S), both encoding an active photopigment (Jagannath et al., 2015) (see above). The study demonstrated that OPN4S mediates light-induced pupillary constriction whereas the OPN4L regulates negative masking. However, both isoforms contribute to light entrainment and light induced sleep induction (Jagannath et al., 2015). The observations show that different splice variants of a single receptor gene can regulate different behaviors.

## Neurotransmitters of the RHT: Functional Significance and Learning From the Knockout Models

Nerve fiber terminals of the RHT are found in the retino-recipient areas of the brain including the SCN, IGL, ventrolateral preoptic nucleus (VLPO) regulating sleep, subparaventicular zone of the PVN, lateral hypothalamic area dorsal to the SON (Hannibal and Fahrenkrug, 2004b; Canteras et al., 2011), and several nuclei in the pretectal area, of which, the most densely innervated is the olivary pretectal nucleus (OPN) controlling the pupillary reflex (Hannibal and Fahrenkrug, 2004b; Hattar et al., 2006; Hannibal et al., 2014). In rat, these projections co-store two neurotransmitters, the classic neurotransmitter glutamate and PACAP (Hannibal et al., 2000; Engelund et al., 2010), targeting several subtypes of glutamate receptors and the PACAP specific receptor, the PAC1 receptor located in the SCN neurons (Hannibal, 2002, 2006b). Glutamate is considered as the primary neurotransmitter having a “light like” phase shifting capacity on the SCN neurons (Hannibal, 2002), while PACAP is considered as a neuromodulator gating the effects of glutamate induced resetting of the circadian phase (Hannibal, 2006b, 2016).

### NIF in Mice Lacking the PACAP or PAC1 Receptor

*In vivo* and *in vitro* studies of the behavior and gene expression using PACAP and PAC1 deficient mice have increased our understanding of the role of PACAP and the PAC1 receptor in NIF (light entrainment, negative masking behavior, and PLR). *In vitro* electrophysiological studies indicate that PACAP can potentiate glutamate induced phase delay during early night and decrease glutamate induced phase advance at late night (Chen et al., 1999; Bergström et al., 2003; Hannibal, 2006b). *In vitro* studies in rat and hamster have provided evidence that PACAP in nanomolar concentrations induces phase shifts similar to light, whereas micromolar concentrations seem to modulate glutamate induced phase shifts (Chen et al., 1999; Harrington et al., 1999). Three independent groups have generated mice lacking the PACAP gene, one group mice lacking the PAC1 receptor gene.

PACAP deficient mice show normal light entrainment although a significant reduced phase shift of the circadian rhythm after a high light intensity stimulation (<100 lux) (Kawaguchi et al., 2003; Colwell et al., 2004). This finding was aligned with the *in vitro* results using PACAP in a micromolar concentration (Chen et al., 1999; Harrington et al., 1999). PACAP KO mice also demonstrated a compromised masking response whereas the PLR seemed unaffected (Kawaguchi et al., 2010). A third strain of PACAP KO mice showed similar free-running periods and normal photoentrainment (Beaule et al., 2009). However, although this strain of PACAP KO mice did not display a phase-advance after single light pulses at late night, the PACAP KO mice did entrain to a 23-h T-cycle (Beaule et al., 2009). In addition, PACAP KO mice needs several LD cycles to re-entrain after a 6-h phase advance of the LD cycle (Beaule et al., 2009). These results indicate that PACAP is required for the normal integration of the phase advancing light signal by the SCN.

PACAP receptor 1 (PAC1) deficient mice showed a normal photoentrainment as the PACAP KO mice, indicating that both genotypes have a stable biological clock (Hannibal et al., 2001a, 2008). PAC1 KO mice entrain to LD cycles but have a significantly reduced response to light stimulation at early subjective night (Hannibal et al., 2008). When placed in T-cycles (circadian day length of 21–26 h), PAC1 KO mice reach the limit of entrainment that is most pronounced at low light intensities (Hannibal et al., 2008). In accordance, PAC1 KO mice significantly needs more time (LD cycles) to re-entrain after an 8-h phase shift of the external LD cycle at low light intensities (jetlag experiment) (Hannibal et al., 2008). Furthermore, the PAC1 KO mice show an impaired negative masking that is most significant at a low light intensity (Hannibal et al., 2008). Together, *in vivo* and *in vitro* studies in mice lacking either PACAP or the PAC1 receptor show that PACAP plays an important role in light regulation of the SCN activity especially at low light intensities.

Light stimulation at early night induces, in mice, large phase delays of the circadian rhythm (see Figure 1) and small phase advances at late night, while in hamsters, light induces large phase advances at late night and small phase delays at early night (Hannibal, 2002). These differences are determined by the length of the endogenous period (tau). Mice, which have a fast running clock (tau shorter than 24 h), need to slow down the clock (phase delay) in order to stay entrained whereas hamsters, having a slow running clock (tau longer than 24 h), need to speed up their clock (phase advance) to stay entrained with the LD cycle (see also section “Light Entrainment”) (Golombek and Rosenstein, 2010). It is possible that the low sensitivity for light in mice at late night explains these phase shift differences at this time point in both the PACAP KO and PAC1 KO mice compared to wild type mice (Golombek and Rosenstein, 2010). The OPN, another NIF target areas which controls the PLR, receives retinal PACAP input and expresses the PAC1 receptor (Engelund et al., 2012). Compared to the wild type mice, the PLR of PAC1 KO mice is significantly attenuated and the difference is significantly increased with higher light intensities (Engelund et al., 2012), indicating a role of the PAC1 receptor signaling in the PLR. However, some discrepancy is found in the PLR comparing the PACAP KO and PAC1 KO mice. It is important to note that although melanopsin in mRGCs are necessary for an intact PLR, mRGCs receive input from the classical photoreceptors (Guler et al., 2008). When studying PLR in PACAP KO mice, they were exposed to blue light [(λ) 460–490 nm] (Kawaguchi et al., 2010). This wave length selectively stimulates the melanopsin photoreceptor (Do and Yau, 2010). PAC1 KO mice were stimulated using white light (Engelund et al., 2012), which also activates rods and cones (Do and Yau, 2010). Recent studies indicate that the PLR is controlled by Brn3b-positive mRGCs (Chen et al., 2011). These mRGCs may be dependent on the input signals from the outer retina (rods and cones) as well as being more sensitive to white light (Chen et al., 2011). Most likely, the melanopsin/PACAP mRGCs that are involved in the regulation of the PLR are different from the mRGCs regulating light entrainment and masking (Chen et al., 2011). The PLR of PAC1 KO is significantly more altered when exposed to light at a higher intensity (Engelund et al., 2012) while entrainment and negative masking in these animals are more affected at a low light intensity (Hannibal et al., 2008), supporting the involvement of the different subtypes of mRGCs.

### NIF in Mice Lacking the Vesicular Glutamate Transporter 2 (VGLUT2) (Glutamate)

The VGLUT2 transporter is important for the process that packs glutamate into synaptic vesicles (Takamori, 2006). The VGLUT2 transporter shows a distinct expression within the CNS and retina (Fujiyama et al., 2003) and both the VGLUT2 (Johnson et al., 2007) and PACAP are co-stored in the melanopsin containing mRGCs (Engelund et al., 2010). Mice with a specific loss of VGLUT2 in mRGCs were investigated at different light intensities (Gompf et al., 2014; Purrier et al., 2014). VGLUT2 KO mice entrained to the LD cycle at 900 lux and all VGLUT2 KO mice showed a normal free-running activity when exposed to constant darkness, indicating an intact circadian clock (Purrier et al., 2014). VGLUT2 KO mice showed a decreased ability to re-entrain after an 8 h shift in the external LD cycle (jetlag experiment) (Purrier et al., 2014), and furthermore, VGLUT2 KO mice failed to entrain to a skeleton photoperiod (1 h of light in the morning and 1 h in the evening, simulating dawn and dusk). These finding suggests that VGLUT2 KO mice had a disturbed setting of signals for dusk and dawn (Purrier et al., 2014). A more severe affected phenotype of the VGLUT2 KO mice was described by Gompf et al. (2014). These mice failed to entrain to the LD cycle (Gompf et al., 2014) and when exposed to light at early night, the VGLUT2 KO mice showed no shift in the circadian phase and no induction of FOS in the SCN, which indicates a strongly compromised light sensitivity in these mice (Gompf et al., 2014). The decreased light sensitivity in these animals is substantiated during constant light conditions. Normal wild type changes their free running tau, which becomes longer than 24 h while the tau of VGLUT2 KO mice remained unchanged during constant darkness (Gompf et al., 2014). When exposing the population of VGLUT2 KO mice to strong constant light (2,000 lux), the tau was increased, indicating that light sensitivity remains, although with a higher threshold in this subpopulation of VGLUT2 KO mice. In both stains of the VGLUT2 KO mice, negative masking was impaired despite the light intensities used (Gompf et al., 2014; Purrier et al., 2014). The PLR was found to be significantly attenuated in VGLUT2 KO mice (Purrier et al., 2014).

The investigations of mice lacking PACAP (PACAP and PAC1 KO mice) and glutamate signaling (VGLUT2 KO mice) confirm the behavior, physiologically and gene expression studies *in vitro* and *in vivo* (reviewed in Hannibal, 2002, 2006b), showing that glutamate and PACAP are the neurotransmitters in the RHT mediating NIF information to the brain. Furthermore, the studies referred to emphasize the role of PACAP as a co-transmitter in the RHT acting together with glutamate, the primary neurotransmitter of the RHT (Hannibal, 2006b). Future studies are needed to clarify the stimulus conditions which release glutamate and PACAP from the RHT nerve terminals due to the stimulation of the photoreceptors (rods, cones, melanopsin). Not only light intensity but also irradiance and wave length (color) are of major importance in the regulation of NIF (Walmsley et al., 2015).

### Photoreceptors and Neurotransmitters Involved in the PLR

Different subtypes of mRGCs are involved in the different NIF functions (Chen et al., 2011; Schmidt et al., 2011a). In a recent study, the PLR was used to investigate the contribution of photoreceptors that initiates light signaling and release of the two neurotransmitters, glutamate and PACAP (Keenan et al., 2016). The study used a combination of mice lacking rods, cones, melanopsin-, PACAP-, and VGLUT2 deficient mice. The results demonstrated a complex interaction between photoreceptors and neurotransmitters in the RHT (Keenan et al., 2016). The transient pupillary response was found to be driven by rod photoreceptors during dim and moderate light intensities. The process was found to be mediated by glutamate and was able to adapt within minutes. The sustained pupillary responses are in contrast, dominated by melanopsin phototransduction in mRGCs and mediated by PACAP, providing a stable pupil maintenance across the day (Keenan et al., 2016). These findings demonstrate how one NIF function (PLR) in the visual system is able to accomplish a high sensitivity, transient, as well as integrative and long-term responses. Other NIF functions such as photoentrainment may be differently regulated and potentially dependent on different subtypes of mRGCs.

## Artificial Light at Night (ALAN) and NIF

**FIGURE 6:**
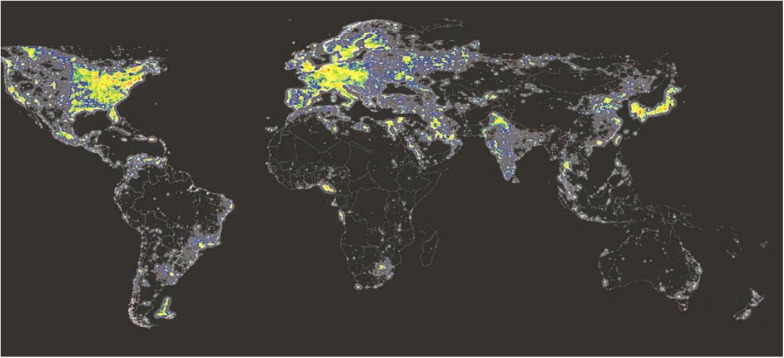
Artificial light at night (ALAN). ALAN levels detected by the US-DMPS satellite sensors in 2010. Note that the areas emitting the highest ALAN levels are marked in red, less lit areas are marked in orange and yellow. Areas with no stable light appear in black (Cinzano et al., 2001). Source: mapped using DMSP (2014) data.

All NIF functions including the circadian timing system have evolved in mammals long before the occurrence of modern society. While many people on earth still lives in rural areas where light is coming primarily from daylight, people in most parts of the industrial world are exposed to artificial white light at night (ALAN) (Figure 6). Electrical white light has changed the lives of humans by extending the time of light during the 24 h solar cycle. This has been an important factor for the industrial growth in our society. However, recent studies have shown that light at night could have a great impact on health, potentially playing an essential role in the development of lifestyle-associated diseases such as metabolic syndrome, sleep problems, and cancer (Navara and Nelson, 2007; Panda, 2016). Since hormone levels vary during the light/dark cycle corresponding to metabolic, reproductive, and immunological functions, ALAN is a potential disruptor of the endocrine functions due to its impact on the circadian regulation and by direct effects of light independent of the circadian system (Hannibal, 2020) (see above section “Masking”). Nocturnal melatonin secretion is the most sensitive neuroendocrine hormone axis to be affected by ALAN (Ouyang et al., 2018). Such disruption in nocturnal melatonin has been associated with an increased risk of developing different forms of cancer (Hansen, 2001; Kloog et al., 2010; Van Dycke et al., 2015). Similarly, a higher occurrence of health problems has been found in people at shift work, such as metabolic syndrome, sleep disturbances, and depression (Shivers et al., 1991; Navara and Nelson, 2007; Fonken et al., 2010).

## Perspectives

Photoentrainment of the circadian clock is fundamental for the stable regulation of neuroendocrine systems leading to the secretion of hormones into the temporal niche controlling physiological functions such as metabolism, sleep, immune systems, and reproduction. Light directly suppresses melatonin secretion independent of the circadian system with an impact on several neuroendocrine axes. In modern societies, ALAN seems to affect NIF including the circadian and neuroendocrine systems. Therefore, it should be taken into serious consideration when trying to gain a better understanding of health problems in the industrialized human population.

## Author Contributions

JH wrote the manuscript.

## Conflict of Interest

The author declares that the research was conducted in the absence of any commercial or financial relationships that could be construed as a potential conflict of interest.
